# Taxonomy, Lectotypification, and Conservation of the Genus *Phyllodium* (Fabaceae: Desmodieae) in Cambodia, Laos, and Vietnam

**DOI:** 10.3390/plants14121822

**Published:** 2025-06-13

**Authors:** Witsanu Saisorn, Jiratthi Satthaphorn, Shuichiro Tagane

**Affiliations:** 1 Department of Biology, School of Science, Walailak University, Nakhon Si Thammarat 80160, Thailand; 2 Center of Excellence for Ecoinformatics, School of Science, Walailak University, Nakhon Si Thammarat 80160, Thailand; jiratthi.sa@wu.ac.th; 3 Kagoshima University Museum, Kagoshima University, Kagoshima 890-0065, Japan; stagane29@gmail.com

**Keywords:** conservation, *Desmodium*, Indochina, IUCN, Leguminosae, Papilionoideae

## Abstract

A taxonomy of the genus *Phyllodium* Desv. in Cambodia, Laos, and Vietnam is presented. The plant specimens collected from the fields and herbarium specimens kept at Asian and European herbaria are examined. The IUCN conservation status of each species at regional and national levels is assessed. Five species are enumerated, viz., *Phyllodium elegans* (Lour.) Desv., *P. kurzianum* (Kuntze) H.Ohashi, *P. longipes* (Craib) Schindl., *P. pulchellum* (L.) Desv., and *P. vestitum* Benth. Lectotypification of two names, *Desmodium longipes* Craib and *D. tonkinense* Schindl., is performed. A key to the species, description, distribution, ecology, phenology, vernacular names, full list of specimens examined, and photographs are provided. The conservation status of five *Phyllodium* species varies across Cambodia, Laos, Vietnam, and Indochina. *Phyllodium elegans* and *P. pulchellum* are consistently Least Concern (LC) in all regions. *Phyllodium kurzianum* is Data Deficient (DD) in Laos, Near Threatened (NT) in Vietnam, and LC in Indochina. *Phyllodium longipes* is EN in Cambodia but LC elsewhere. *Phyllodium vestitum* is NT in Cambodia, Vulnerable (VU) in Laos and Vietnam, and LC in Indochina. The taxonomic information provided in this work will contribute to the advancement of the Flora of Cambodia, Laos, and Vietnam and the conservation status of each species proposed in this paper can be used for future conservation planning.

## 1. Introduction

The genus *Phyllodium* belongs to the family Fabaceae and subfamily Papilionoideae. It was first described by Desvaux [1], based on two names, *Phyllodium pulchrum* Desv. (=*P. pulchellum* (L.) Desv.) and *P. lutescens* (Poir.) Desv. (=*Pycnospora lutescens* (Poir.) Schindl.). The genus was previously treated under either *Desmodium* Desv. or *Dicerma* DC. by subsequent studies, at least at three different taxonomic ranks, viz., *Dicerma* sect. *Phyllodium* (Desv.) Benth., *Desmodium* sect. *Phyllodium* (Desv.) Benth., and *Desmodium* subgen. *Phyllodium* (Desv.) Baker [2,3,4]. However, at least three taxonomic treatments recognized *Phyllodium* as a distinct genus due to its primary bracts and phylogenetic analyses [5,6,7]. *Phyllodium* was divided into two subgenera by Ohashi [8] based on primary bracts, dimorphic (subgen. *Prainia* H.Ohashi) or uniform (subgen. *Phyllodium*). Currently, the genus comprises seven recognized species, namely *P. elegans* (Lour.) Desv., *P. hackeri* Pedley, *P. insigne* Schindl., *P. kurzianum* (Kuntze) H.Ohashi, *P. longipes* (Craib) Schindl., *P. pulchellum*, and *P. vestitum* Benth [8,9,10].

*Phyllodium* is classified under the tribe Desmodieae based on its articulate pod [5]. Phylogenetic analyses utilizing DNA regions such as *rbc*L, *psb*A-*trn*H, and ITS-1 provide additional evidence supporting its classification within this tribe. Moreover, the ITS-1 analysis indicates that *Phyllodium* is closely related to *Dendrolobium* (Wight & Arn.) Benth [6,7]. The genus can be distinguished from other genera within the tribe by the presence of foliaceous primary bracts, which are typically distributed along the entire inflorescence axes in most species. However, in *P. insigne*, the only species of subgen. *Prainia*, this type of bract is restricted to the lower portion of the inflorescence [8,10].

The genus is distributed across tropical and subtropical regions of Asia. One species, *P. pulchellum*, exhibits a broad geographic range extending from Asia to northern Australia, whereas *P. hackeri* is endemic to Queensland, Australia [6,9,10,11,12]. The genus has been reported from several countries, including India (1 sp. [13]), Sri Lanka (1 sp. [14]), Bhutan (1 sp. [15]), China (4 spp. [16]), Laos (5 spp. [17,18]), Thailand (6 spp. [10]), the Philippines (1 sp. [19]), and Australia (ca. 4 taxa [9]). Additionally, the genus has been recorded in several regions such as Indochina (5 spp. [20]), the Malay Peninsula (2 spp. [21]), Java (2 spp. [22]), Malesia (3 taxa [11]), and New Guinea (1 sp. [23]). However, the taxonomic status and species number of *Phyllodium* in Cambodia, Laos, and Vietnam remains ambiguous due to synonyms, typification, and a lack of taxonomic information updates since 1994. Therefore, this work aims to (1) provide taxonomic details of the genus for these countries and revise the key to species, (2) designate lectotypes by following the International Code of Nomenclature for algae, fungi, and plants (ICN) [24], as well as (3) assess the conservation status of each species at regional and national levels.

## 2. Results

A total of five *Phyllodium* species are recorded in Cambodia, Laos, and Vietnam, including *Phyllodium elegans*, *P. kurzianum*, *P. longipes*, *P. pulchellum*, and *P. vestitum*. They are shrubs with trifoliolate and alternate leaves, two free stipules, and two stipels. Inflorescences are of the pseudoracemose type with foliaceous primary bracts and a secondary bract. Flowers are subtended by two bracteoles and consist of campanulate calyx, five petals, 10 monadelphous stamens, and a superior ovary. Pods are loments with 1–7 articles. The key to the species of *Phyllodium* is provided as follows:

**Key to the species**

1. Pods with puberulous hairs…………………………………………………………….**2**

1. Pods with silvery appressed hairs…………………………………………**1. *P. elegans***

2. Pedicels less than 7 mm long……………………………………………………………**3**

2. Pedicels 7–10 mm long…………………………………………………**2. *P. kurzianum***

3. Flowers shorter than 10 mm……………………………………………………………**4**

3. Flowers longer than 10 mm………………………………………………**5. *P. vestitum***

4. Flowers less than 10 per fascicle………………………………………**4. *P. pulchellum***

4. Flowers equal to 10 or more than 10 per fascicle…………………………**3. *P. longipes***

**Figure 1:**
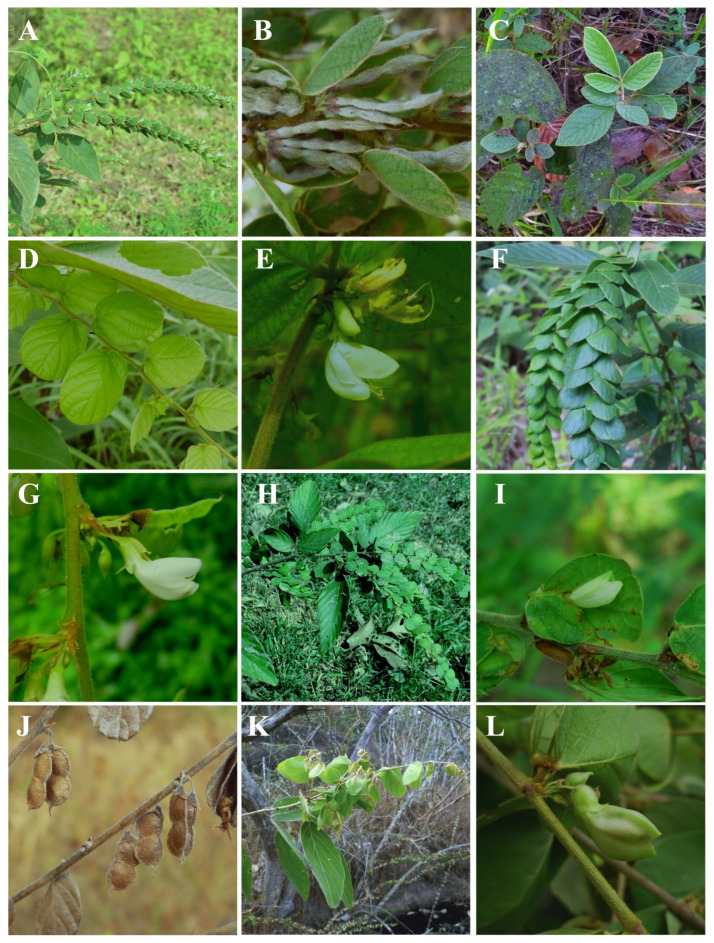
Morphological characteristics of *Phyllodium*. (**A**,**B**). *P. elegans*: (**A**). inflorescence and (**B**). fruits; (**C**–**E**). *P. kurzianum*: (**C**). leaves, (**D**). foliaceous primary bracts, and (**E**). flowers; (**F**,**G**). *P. longipes*: (**F**). inflorescence and (**G**). flowers; (**H**–**J**). *P. pulchellum*: (**H**). inflorescence, (**I**). flowers, and (**J**). fruits; (**K**,**L**). *P. vestitum*: (**K**). inflorescence and (**L**). flowers. Photographs: (**A**) by Teerawat Srisuk and (**F**) by Yotwarit Phansenee.

**Figure 2:**
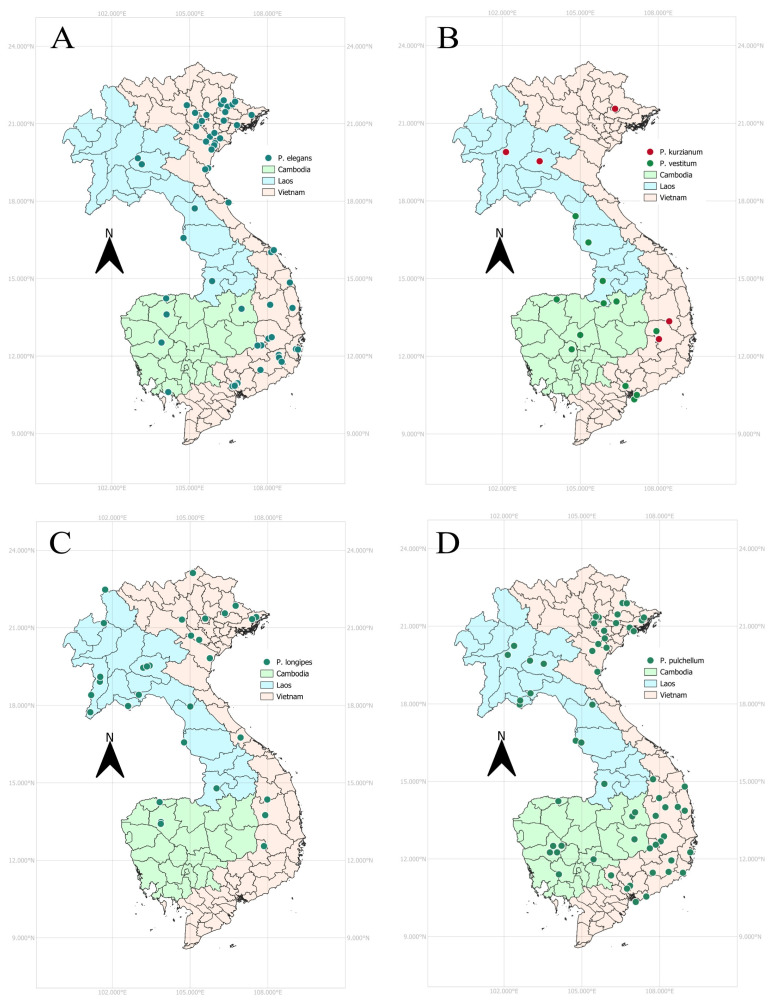
Distribution maps of *Phyllodium* in Cambodia, Laos, and Vietnam. (**A**). *P. elegans*; (**B**). *P. kurzianum* and *P. vestitum*; (**C**). *P. longipes*; (**D**). *P. pulchellum*. Maps drawn using QGIS software version 3.34.14.

**1. *Phyllodium elegans*** (Lour.) Desv. (1826: 324). Type: China, Canton and Macao, Jan. 1837, *M.Gaudichaud 147* (neotype **P!** P00093020, designated by Dy Phon et al. [20], isoneotype **G!** G00020060). Figure 1A,B and Figure 2A.

*Hedysarum elegans* Lour. (1790: 450).

*Zornia elegans* (Lour.) Pers. (1807: 318).

*Dicerma elegans* (Lour.) DC. (1825: 339).

*Desmodium elegans* (Lour.) Benth. (1861: 83).

*Meibomia elegans* (Lour.) Kuntze (1891: 198).

*Phyllodium elegans* var. *typicum* Schindl. (1924: 270).

*P. elegans* (Lour.) Desv. var. *javanicum* Schindl. (1924: 270). Type: Indonesia, Java, *Horsfield 81* (Lectotype **CAL**, n.v., designated by Ohashi et al. [25], isolectotypes **GH!** 00967182 (digital image), **K!** K001235161).

*Desmodium blandum* van Meeuwen (1962: 247).

Shrubs, up to 4 m tall with tomentose hairy throughout; stipules 3–6 mm long; petioles 1–2.5 mm long; rachis up to 2 cm long. *Leaflets* lanceolate 3–12 cm long, 1–6 cm wide. Inflorescences up to 0.5 m long; foliaceous primary bracts present; secondary bracts ovate, up to 1 mm long. *Flowers* 5–8 mm long, 7–15 per fascicle; bracteoles linear, 0.9–1 mm long; pedicels 1.5–3 mm long; calyx tube 1–1.5 mm long; petals white, 5–7 mm long; stamens up to 7 mm long. *Pods* 2–4 articulate, up to 2.5 cm long, outer surface smooth, silvery appressed hairy; articles 3–3.5 mm long; fruit stipe 2–4 mm long; seed dark brown, reniform, 2.5–3 mm long, 1.5–2.2 mm wide.

**Distribution**—China, Myanmar, Thailand, Laos, Cambodia, Vietnam, and Indonesia.

**Ecology**—Open forest and grassland of degraded areas on sandy or clay soils, 30–2170 m alt.

**Phenology**—Flowering: August–May. Fruiting: August–May.

**Vernacular**—CAMBODIA: ben eui (Khmer-Siem Reap); kantrum pre kraoy (Khmer-Pursat); kantuy trakuet, sraka: bangkuey. VIETNAM: cay vang rong (Lang Son); ro sar tai (Lam Dong); vey te te (Ha Nam).

**Specimens examined**—CAMBODIA: **Kampot** [27 September 1903, *Geoffray 107* (**P**); *L.Hahn 40* (**P**); 1927, *L.Hahn 42* (**P**)]; **Pursat** [Prey Smach pres de Leach, 5 October 1965, *M.A.Martin 43* (**P**)]; **Ratanak Kiri** [Veal Thmor Longley, 13°49.994′ N 107°00.108′ E, 306 m alt., 22 November 2007, *B.David* et al. *CL712* (**K**, **P**)]; **Siem Reap** [Phnom Kulen, 100 m alt., 15 December 1968, *M.Martin 1316* (**P**-2 sheets); Pied massif Dongrek, entre Samrong et Anlong Veng, 30 October 1927, *Poilane 13821* (**P**-2 sheets)]; **no province specified** [Pen Lovier, March 1870, *L.Pierre 1004* (**P**); Plantes du Cambodge, *M.Poree-Maspero 328* (**P**); expedition du Me-Kong, 1866–1868, *Thorel s.n.* (**P**)]. LAOS: **Xiengkhuang** [Phonsavan district, Plain of Jars I, 6 September 2017, *C.Maknoi* et al. *L10-035* (**QBG**); Tran Ninh, 1160 m alt., November 1931, *Petelot s.n.* (**P**); Tran Ninh, Phoun Phiang, 1600 m alt., November 1931, *Petelot s.n.* (**P**)]; **Khammouane** [Inundation zone, near Nakai, 17°43′14″ N 105°11′52″ E, 547 m alt., 7 November 2005, *M.F.Newman* et al. *LAO 955* (**NUOL**)]; **Savannakhet** [13 October 1938, *Poilane 28038* (**P**-2 sheets)]; **Champasack** [Bassac, expedition du Me-Kong, 1866–1868, *Thorel s.n.* (**P**)]. VIETNAM: **Yen Bai** [25 November 1913, *A.Chevalier 29143* (**P**-2 sheets)]; **Phu Tho** [April 1925, *P.A.Petelot & Du Pasquier s.n.* (**P** P00093076)]; **Vinh Phuc** [Nong Truong Tam Dao, 11 August 1963, *T.Vy s.n.* (**HNU**)]; **Lang Son** [Cao Loc, Tan Thanh, 29 August 1963, *Dai s.n.* (**HNU**); Van Linh, *Eberhardt 3287* (**P**); Deo Sai Ho, 20 November 1961, *N.D.Khoi 1027* (**HNU**); Nuoc Binh, 21 October 1911, *H.Lecomte & A.Finet 378* (**P**); Huu Lung, Hoa Thang, 19 October 1963, *T.Vy s.n.* (**HNU**)]; **Quang Ninh** [Ouonbi au nord de Quang Yen, 12 September 1885, *B.Balansa 1251* (**K**, **P**); Kau Nga Shan and vicinity, Tien Yen, 23 September–7 October 1940, *W.T.Tsang 30482* (**C**, **E**, **K**, **P**, **SING**)]; **Hai Duong** [Sept Pagodes, September 1906, *M.Mouret 40* (**P**)]; **Hoa Binh** [Mont Bavi, 5 April 1909, *Ch.d’Alleizette s.n.* (**P**); Ha Son Binh, Lac Thuy, 22 October 1964, *N.D.Ngoi s.n.* (**HNU**-2 sheets); Ha Son Binh, Tu Ly, 20 November 1980, *N. van Trep NT-1197* (**HNU**); ibid., 21 November 1950, *N. van Trep NT-1199* (**HNU**-7 sheets)]; **Ha Nam** [Yen Bac, 19 October 1888, *H.Bon 4036* (**P**-4 sheets); Ha Nam Ninh, Nong Truong Dong Giao, 9 August 1962, *N.Q.Hao s.n.* (**HNU**)]; **Ninh Binh** [Cho Ganh, 28 March 1914, *M.Duport 43* (**P**-2 sheets); ibid., September 1923, *Petelot 1038* (**C**, **P**-2 sheets, **SING**); ibid., October 1922, *Petelot 1107* (**P**-2 sheets); ibid., October 1922, *Petelot 1707* (**P**); Dong Co, Bac Vangm Dong Giao, 9 August 1962, *Q.Hao 3160* (**HNU**); Nho Quan, Cuc phuong, 22 October 1962, *N.D.Khoi 405* (**HN**); ibid., 27 October 1963, *N.D.Khoi s.n.* (**HN**-2 sheets); Cuc phuong, 27 October 1963, *N.D.Khoi 812* (**HN**); ibid., 27 October 1963, *N.D.Khoi 813* (**HN**); Dong Giao, Cho Ganh, October 1962, *S.N. 3160* (**HNU**)]; **Thanh Hoa** [Ha Trung, Nong Truong, 27 August 1963, *N.D.Ngoi s.n.* (**HNU**); Annam, Hoang Mai, November 1932, *Rothe 91* (**P**)]; **Nghe An** [Quynchau, 19°14′ N 105°36′ E, 100 m alt., 6 December 1993, *R.Schultze-Kraft* et al. *059/012-02* (**K**)]; **Ha Tinh** [Ha Tinh-Hue, 17°57′ N 106°30′ E, 150 m alt., 6 December 1993, *R.Schultze-Kraft* et al. *059/016-02* (**K**)]; **Quang Tri** [Annam, col d’ Ai Lao, 400 m alt., 1 September 1927, *Poilane 13578* (**AAU**, **P**); Annam, Hon Rao, 500 m alt., 12 October 1939, *Poilane 30085* (**P**)]; **Thua Thien Hue** [Nui Bach Ma, 1400 m alt., 14 April 1939, *Poilane 29661* (**P**)]; **Quang Ngai** [Pho Van, Pho Van-Van Ly, 14°51′ N 108°52′ E, 40 m alt., 31 May 1993, *R.Schultze-Kraft* et al. *059/026-06* (**K**)]; **Binh Dinh** [Qui Nhon, Qui Nhon-Pleiku, 13°52′ N 108°58′ E, 150 m alt., 22 July 1993, *R.Schultze-Kraft* et al. *059/029-07* (**K**)]; **Gia Lai** [Dak Doa, Mang Yang, 750 m alt., 26 October 1978, *P.K.Loc & N.Ba P-2596* (**HNU**-3 sheets)]; **Khanh Hoa** [Annam, Nha Trang, 4 March 1922, *Poilane 2686* (**P**-2 sheets); Massif de Co Inh pres Nha Trang, 18 September 1922, *Poilane 4648* (**P**); Dong Bo pries de Nha Trang, 30 m alt., 15 February 1923, *Poilane 5526* (**P**); Merre et l’Enfant, 300 m alt., 11 November 1922, *Poilane 5132* (**P**); Nha Trang and vicinity, 11–26 March 1926, *C.B.Robinson 1277* (**K**, **P**)]; **Dak Lak** [Region de Ban Me Thuot, 17 December 1948, *Schmid 756* (**P**); Ban Me Thuot, 12°44′ N 108°10′ E, 250 m alt., 10 November 1993, *R.Schultze-Kraft* et al. *059/036-02* (**K**); Ban Me Thuot, Ban Me Thuot-Ho Chi Minh, 12°25′ N 107°46′ E, 800 m alt., 30 September 1993, *R.Schultze-Kraft* et al. *059/039-01* (**K**)]; **Dak Nong** [Dak Mil, Duc Minh, 9 December 1979, *H.Dung 424* (**HN**-3 sheets)]; **Lam Dong** [Dran, 1000 m alt., 3 May 1919, *A.Chevalier 40540* (**P**); Bac Thai, Bac Son, 13 October 1963, *M.Duc 13* (**HNU**); Da Lat, Ang Kroet pres Dankia, 26 October 1920, *F.Evrard 372* (**P**-2 sheets); Lang Bian, 14 October 1924, *F.Evrard 1437* (**P**-2 sheets); Da Lat, Lang Hanh, 800 m alt., 19 September 1965, *M.A.Martin 951* (**P**); Annam, Haut Donai, stationed Lang Hanh, 1000–1200 m alt., 4 October 1932, *Poilane 21081* (**P**); ibid., 1000 m alt., 8 October 1932, *Poilane 21164* (**P**); Annam, Haut Donai, station Agricole de Blao, 800 m alt., 1 March 1933 *Poilane 22166* (**AAU**, **P**); Annam, Haut Donai, 119 km de la route col. no. 20, 700–800 m alt., 22 October 1932, *Poilane 21228* (**P**); Annam, Haut Donai, pied du Bran pres de Djiring, 5 March 1935, *Poilane 24741* (**AAU**, **BKF**, **P**); Da Lat, village de Teurnoum, 1959, *P.Tixier s.n.* (**P**); Din Duong, 1500 m alt., 2 August 1983, *S.N. 1146* (**HN**)]; **Dong Nai** [Bien Hoa, 200–800 m alt., 10 January 1914, *Henry 29885* (**P**); Bien Hoa, 1862–1866, *Thorel 722* (**BM**, **E**, **P**-5 sheets)]; **Ha Noi** [Son Tay, Bavi, 9 January 1965, *D. KS Viet-Trung s.n.* (**HN**-2 sheets)]; **Da Nang** [Tourane and vicinity, 30 July 1927, *J. & M.S.Clemens 3995* (**P**); Pied montagne, Ba Na et Tourane, 10 March 1939, *Poilane 29330* (**P**)]; **Ho Chi Minh** [Cochinchine, Saigon, *M.Germain 105* (**P**); Plane des Tombeaux, pres Saigon, 1 November 1864, *E.Lefevre 41* (**P**); Point A, route de Saigon a Bien Hoa, 22 November 1864, *E.Lefevre 322* (**P**); Saigon, Thu Duc, August 1867, *L.Pierre 221* (**BM**, **E**-2 sheets, **K**, **P**-2 sheets); ibid., August 1867, *L.Pierre s.n.* (**P**); **Tonkin** [Tu Phap, October 1887, *B.Balansa 2189* (**K**, **P**-2 sheets); Huong Kahn, February 1909, *S.N. 14bis* (**P**); ibid., 1908, *S.N. 106* (**P** P03031729)]; **no province specified** [October 1882, *Jardin de Saigon Staff s.n.* (**P** P00093071); Boa-Loc, Prenn-Tha, ca. 1050 m alt., 28 December 1997, *C.Phengklai* et al. *10686* (**QBG**); 28 August 1963, *D.M.Thai s.n.* (**HNU**)].

**Conservation status**—CAMBODIA: The collections were recorded from various locations across Cambodia, covering a large Extent of Occurrence (EOO) of 68,112.06 km^2^ and an Area of Occupancy (AOO) of 20 km^2^. As a result, this species is assessed as Least Concern (LC) in the country. LAOS: The specimens were collected from three regions of Laos. With an EOO of 31,539.31 km^2^ and an AOO of 20 km^2^, the species is proposed to be assessed as LC. VIETNAM: The species has been found in several regions of Vietnam, with an EOO of 327,508.52 km^2^ and an AOO of 192 km^2^. Therefore, it is assessed as LC. INDOCHINA: At the regional level, this species is assessed as LC.

**2. *Phyllodium kurzianum*** (Kuntze) H.Ohashi (1973: 272). Figure 1C–E and Figure 2B.

*Meibomia kurziana* Kuntze (1891: 197).

*Desmodium grande* Kurz (1874: 184). Type: Myanmar, Tagoung, *Anderson* 168 (isotype **CAL** n.v.).

*Phyllodium grande* (Kurz) Schindl. (1924: 270).

*Desmodium kurzii* Craib (1911: 37). Type: Thailand, Chiang Mai, in the deciduous jungle on Doi Suthep, 300 m, *A.F.G. Kerr* 766 (holotype **K!** K000587486).

*Phyllodium kurzii* (Craib) Chun (1940: 213).

Shrubs, up to 2 m tall, puberulous to tomentose; stipule up to 5 mm long; petioles 2.5–6 mm long; rachis 2–4 cm long. *Leaflets* lanceolate, ovate, or elliptic, 5–18 cm long, 2.5–11 cm wide. Inflorescences up to 0.5 m long; foliaceous primary bracts present; secondary bracts ovate, up to 0.7 mm long. *Flowers* up to 10 mm long, 10–15 per fascicle; bracteoles ovate, 0.5–0.8 mm long; pedicels up to 10 mm long; calyx tube 1.5–2 mm long; petals white to light yellow, 7–9 mm long; stamens up to 10 mm long. *Pods* 1–5 articulate, up to 1.5 cm long, outer surface reticulate, puberulous; articles rectangular to suborbicular, 4–5 mm long; fruit stipe 6–7 mm long; seeds brown, reniform, 2.5–3.5 mm long, 2.5–3 mm wide.

**Distribution**—China, Myanmar, Thailand, Laos, and Vietnam.

**Ecology**—Commonly found in a pine–oak forest and dry dipterocarp forest mixed with pine trees, 150–1010 m alt.

**Phenology**—Flowering: July–December. Fruiting: September–April.

**Vernacular**—LAOS: ko ket linh (Xiengkhuang).

**Specimens examined**—LAOS: **Luang Prabang** [Plantes du Laos, 30 April 1895, *M.Massie s.n.* (**P**-4 sheets, P00093111, P00093112, P00093113, and P00093114)]; **Xiengkhuang** [Traninh, environs de Xieng Khouang, September–December 1917, *Chevalier 37209* (**P**); Traninh, 15 November 1920, *Poilane 2355* (**AAU**-2 sheets, **P**-3 sheets)]. VIETNAM: **Lang Son** [Huu Lung, Tam Thanh, 12 October 1964, *C.Sam 93* (**HNU**)]; **Dak Lak** [Hau Bon (Cheo Reo), *J.Dournes s.n.* (**P** P02937540); Ban Me Thout, *Schmid s.n.* (**P**-2 sheets, P00093118, and P00093119)].

**Conservation status**—LAOS: A few specimens were collected from fewer than five locations in northern Laos. The species has a limited geographic range, with an EOO of 206.73 km^2^ and an AOO of 12 km^2^. As a result, the species should be assessed as Endangered (EN). However, growing at an altitude between 150 and 1010 m above sea level could suggest that this species prefers various ecologies and may have several populations. Thus, it could be assessed as Data Deficient (DD) instead. VIETNAM: This species has an EOO of 27,964.67 km^2^ and an AOO of 12 km^2^. With fewer than five known populations and a fragmented geographic range, the species is assessed as Near Threatened (NT) for Vietnam. INDOCHINA: Although this species has relatively few recorded collections compared to other species, its EOO and AOO values are sufficiently large to support an assessment of LC for the region.

**3. *Phyllodium longipes*** (Craib) Schindl. (1924: 270). Figure 1F,G and Figure 2C.

*Desmodium longipes* Craib (1910: 20). Type: Thailand, mixed jungle at foot of Doi Sutep, 10 July 1909, *A.F.G. Kerr* 715 (first-step lectotype **K**, designated by Dy Phon et al. [20]; second-step lectotype **K!** K000587485 (https://shorturl.at/N6lP0, accessed on 10 January 2025), designated here; isolectotypes **ABD!**, **BM!** BM000839658, **K!**, **TCD!** TCD0015838, digital image).

*D. tonkinense* Schindl. (1916: 53). Type: Vietnam, Tonkin, Ouonbi, 12 September 1885, *B.Balansa* 1252 (lectotype **P!** P00093141 (https://shorturl.at/TfoBg, accessed on 10 January 2025), designated here; isolectotypes **BR!** BR000000517124, digital image and **BR!** BR000000517156, digital image, **G-DC!**, **G-DC!**, **K!**).

Shrubs, up to 3 m tall with puberulous to pubescent hairy; stipules 3–5 mm long; petioles 2–5 mm long; rachis 0.5–2 cm long. *Leaflets* lanceolate, ovate, or narrowly elliptic, 1–25 cm long, 1–10 cm wide. Inflorescences 0.1–1.5 m long, multi-branched; foliaceous primary bracts present; secondary bracts ovate, up to 1 mm long. *Flowers* 6–9 mm long, 10–25 per fascicle; bracteoles ovate, up to 1 mm long; pedicels 3–5 mm long; calyx tube 1.2–2 mm long; petals white, 6–9 mm long; stamens 6–9 mm long. *Pods* 2–7 articulate, 1.2–2.5 cm long, outer surface reticulate, puberulous; articles rectangular to suborbicular, 3–5 mm long; fruit stipe 4–6 mm long; seeds brown, suborbicular to reniform, 1.2–3.3 mm long, 2–2.2 mm wide.

**Distribution**—China, Myanmar, Thailand, Cambodia, Laos, and Vietnam.

**Ecology**—Grassland, semi-shaded areas at the edge of the forest, and mixed deciduous forest, 170–1025 m alt.

**Phenology**—Flowering: September–July. Fruiting: August–February.

**Vernacular**—LAOS: ket lin (Vientiane), ket pa (Xayabury).

**Specimens examined**—CAMBODIA: **Siem Reap** [Angkor Thum, 1875, *Godefroy 664* (**P**); Angkor Wat, July 1875, *Godefroy s.n.* (**P** P00093126); Angkor Wat, July 1875, *L.Pierre s.n.* (**P** P00093128); Entre Samrong et Anlong Veg pre de la chaine des Dongrek, 30 October 1927, *Poilane 13857* (**P**-2 sheets)]. LAOS: **Vientiane** [Tadluk, Phou Khao Khuay National Biodiversity Conservation Area, 20 November 2005, 170 m alt., *K.Chanthavongsa 25* (**BKF**); Environs de Vientiane, 23 October 1955, *P.Tixier 17* (**P**); That Luang, 25 January 1955, *P.Tixier s.n.* (**P** P00093136); Ban Sa Phan Mo, 5 February 1950, *J.E.Vidal 1171B* (**P**)]; **Phongsaly** [Yod Ou district, Lantouy International Checkpoint, 22.476343° N 101.719168° E, 1025 m alt., *W.Tanming* et al. *L14-286* (**QBG**)]; **Xayabury** [Ban Nam Pouy, 6 September 2015, *C.Maknoi* et al. *L4-150* (**QBG**); Boten, Ban Muang Ham, 7 September 2015, *C.Maknoi* et al. *L4-236* (**QBG**); Boten, Ban Muang Phae, 8 September 2015, *C.Maknoi* et al. *L4-317* (**QBG**); Boten, Lhang Pha Daeng, 9 September 2015, *C.Maknoi* et al. *L4-427* (**QBG**); Boten, Ban Muang Ram, Pha Dang mountain, 17.7384° N 101.1482° E, 780–900 m alt., *C.Maknoi* et al. *L18-049* (**QBG**); Thongmixai, Ban Thad, Nam Sing river, 18.40284 N 101.18097 E, 575–620 m alt., *C.Maknoi* et al. *L18-198* (**QBG**)]; Phiang, Nambouy village, 450 m alt., 31 August 1999, *J.F.Maxwell 99-267* (**CMUB**); Boten, Phu Pha Daeng, 17°44′16.4″ N 101°08′50.2″ E, 773 m alt., 20 February 2019, *W.Thammarong* et al. *L15-028* (**QBG**); **Xiengkhuang** [Tran Ninh, September–December 1917, *Chevalier 37123* (**P**); ibid., September 1930, *Petelot 4628* (**P**); Phonsavan district, Ban Kang Kai, 1100 m alt., 6 September 2017, *C.Maknoi* et al. *L10-112* (**QBG**); Plantes du Laos, *Spire 407* (**P**)]; **Bolikhamxay** [Vieng Thong, Ban Hin Ngon, 460 alt., 18.3777° N 104.4455° E, 3 September 2023, *S.Tagane et al. Z94* (**KAG**)]; **Khammouane** [Khet Sap Hia, 17°57′34″ N 105°0′19″ E, 544 m alt., 27 May 2006, *M.F.Newman* et al. *LAO 1326* (**FOF**, **NUOL**, **P**)]; **Savannakhet** [13 October 1938, *Poilane 28030* (**P**)]; **Champasack** [Pathumphon, Xe Pian NBCA, 13°31′ N 106°21′ E, 25 January 1997, *J.Klackenberg 1080* (**AAU**, **P**)]. VIETNAM: **Son La** [Phu Yen, Muong Thai, Thai Ha village, 21°18′58.7” N 104°41′10.8” E, 6 October 2008, *N.V.Du* et al. *HNK2553* (**K**-2 sheets); ibid., 21°19′0.4” N 104°41′8.8” E, 6 October 2008, *N.V.Du* et al. *RPC77* (**K**)]; **Ha Giang** [Yen Minh district, 23°07′19.0″ N 105°06′29.1″ E, 789 m alt., 11 July 2019, *W.Pongamornkul* et al. *VN2 65* (**QBG**)]; **Vinh Phuc** [Huong Kahn, February 1909, *Ch.d’Alleizette 14* (**P**); Huong Kahn, October 1908, *Ch.d’Alleizette s.n.* (**P** P00093139)]; **Lang Son** [Doc Co Xa Dong Tan, Huu Lung, 14 November 1962, *Bang 1028* (**HNU**); Huu Lung, Minh Son, 13 June 1962, *Dao s.n.* (**HNU**); Huu Lung, November 1962, *N.D.Khoi 1028* (**HNU**-2 sheets); ibid., 29 August 1967, *D.M.Thai 140* (**HNU**); ibid., 12 November 1962, *S.N. 1028* (**HNU**); ibid., 14 November 1962, *S.N. 1028* (**HNU**); ibid., 13 June 1962, *Lun s.n.* (**HNU**)]; 19 November 1962, *N.D.Khoi s.n.* (**HN**-2 sheets); Nuoc Binh, 21 October 1911, *H.Lecomte & A.Finet 336* (**P**); Huu Lung, Don Tam, 4 June 1964, *D.X.Sam s.n.* (**HNU**); **Quang Ninh** [Mon Cay, Tien Yen, 27 August 1932, *Rothe 37* (**P**); Kau Nga Shan and vicinity, Tien Yen, 13–22 December 1936, *W.T.Tsang 27355* (**C**, **E**, **K**, **P**); Sai Wong Mo Shan, Long Ngong village, Dam Ha, 18 July–9 September 1940, *W.T.Tsang 30210* (**C**, **K**, **P**); Ho Yung Shan and vicinity, Tien Yen, 13 September–7 October 1940, *W.T.Tsang 30492* (**K**, **P**, **SING**); ibid., 13 October–22 November 1940, *W.T.Tsang 30677* (**K**, **P**, **SING**)]; **Hoa Binh** [Mai Chau, 10 April 1963, *L.D.Man 862* (**HN**-2 sheets); Hoa Binh, Hoa Binh-Nho Quan, 20°32′ N 105°21′ E, 80 m alt., 22 July 1993, *R.Schultze-Kraft* et al. *059/003-03* (**K**)]; **Thanh Hoa** [Phu Luong-Ba Thuot, 21 November 2020, *T.T.Bach 5757* (**HN**)]; **Quang Tri** [Col d’Ailao, 400 m alt., 1 September 1927, *Poilane 13579* (**P**-2 sheets)]; **Kon Tum** [Dak To, 1000 m alt., 23 September 1930, *Poilane 18365* (**P**-2 sheets)]; **Gia Lai** [Chu Prong, 23 October 1978, *P.K.Loc & N.H.Ha P-2352* (**HNU**-4 sheets)]; **Dak Lak** [Ban Me Thuot, Ban Me Thuot-Ho Chi Minh, 12°33′ N 107°51′ E, 400 m alt., 21 January 1994, *R.Schultze-Kraft* et al. *059/038-02* (**K**)]; **Cochinchine** [Plants of Cochinchine, 4 February 1877, *Godefroy s.n.* (**K**)].

**Conservation status**—CAMBODIA: This species is classified as EN: B2ab(iv) because only a few specimens have been recorded, all from the northern region of Cambodia. It exhibits a small EOO of 46.41 km^2^ and a restricted AOO of 12 km^2^. LAOS: This species is distributed across multiple regions of Laos and is therefore assessed as LC for the country. It has an EOO of 181,547.87 km^2^ and an AOO of 60 km^2^. VIETNAM: Several specimens of this species were collected from the northern, central, and southern regions of Vietnam. The species has an EOO of 186,609.03 km^2^ and an AOO of 56 km^2^, resulting in LC status. INDOCHINA: This species is classified as LC.

**4. *Phyllodium pulchellum*** (L.) Desv. (1813: 124). Figure 1H–J and Figure 2D.

*Hedysarum pulchellum* L. (1753: 747). Type: India, Herb. Linn. No. 921.24 (lectotype **LINN!**, digital image, designated by Dy Phon et al. [20]).

*Dicerma pulchellum* (L.) DC. (1825: 339).

*Desmodium pulchellum* (L.) Benth. (1861: 83).

*Meibomia pulchella* (L.) Kuntze (1891: 197).

*Zornia pulchella* (L.) Pers. (1807: 318).

Shrubs, 0.5–5 m tall with puberulous hairy; stipules 4–7 mm long; petioles 0.3–2 mm long; rachis 0.5–3 cm long. *Leaflets* lanceolate to ovate, 1–20 cm long, 0.5–8 cm wide. Inflorescences 0.1–1 m long, multi-branched; foliaceous primary bracts present; secondary bracts ovate, up to 1 mm long. *Flowers* 5–9 mm long, 4–6 per fascicle; bracteoles up to 1.2 mm long; pedicels 2.5–4 mm long; calyx tube 1–1.5 mm long; petals creamy white, 5.5–8.5 mm long; stamens 6–8 mm long. *Pods* 2–3 articulate, 6–10 mm long, outer surface reticulate, puberulous, both suture fimbriate; articles rectangular to suborbicular, 3.5–4.5 mm long; fruit stipe 1–4.5 mm long; seeds dark brown, rectangular to reniform, ca. 2 mm long, ca. 1.8 mm wide.

**Distribution**—India, Sri Lanka, China, Myanmar, Japan, Taiwan, Thailand, Cambodia, Laos, Vietnam, Malesia, and northern Australia.

**Ecology**—In an open mixed deciduous forest, open area with the grass of dry dipterocarp forest, secondary forest with bamboo domination, edge of the forest, and nearby the river, growing on sandy, clay soil or a rocky area, 15–1200 m alt.

**Phenology**—Flowering: July–March. Fruiting: July–February.

**Vernacular**—CAMBODIA: ang prom pre kroy (Mondul Kiri, Pursat); am prom pre kroi kraham (Kampong Cham); am prom pre kroi sar (Kampong Cham); jrao cati (Ratanak Kiri); kom prum pre kroy (Pursat); teptelr (Mondul Kiri). LAOS: ket lin (Louang Prabang); ket lin noi (Vientiane, Xieng Khouang); ko hang lin (Xieng Khouang); lin noi (Louang Prabang). VIETNAM: cay tu bi (Ba Ria-Vung Tau); rok togla (Dak Lak); sor ti (Lam Dong); tan san (Ba Ria-Vung Tau); xa tay bi ne (Khanh Hoa).

**Specimens examined**—CAMBODIA: **Kampong Cham** [18 July 1930, *Bejaud 16* (**P**); 18 July 1930, *Bejaud 16bis* (**P**); Chup res Kampong Cham, November 1921, *F.Evrard 737* (**P**-2 sheets)]; **Kampong Speu** [Kirirom, 24 November 1999, *S.Hul* et al. *778* (**P**)]; **Kompot** [Mt. Bokor, 15 m alt., 21 October 2012, 10°34′ 29.51” N 104°03′ 57.64” E, *T.Yahara* et al. *4372* (**FU**)]; **Mondul Kiri** [18 December 2015, *S.Hul* et al. *5081* (**P**); Sen Monorom, 12°30′40.7″ N 104°12′57.2″ E, 630 m alt., 1 November 2016, *S.Tagane* et al. 7120 (**FU**)]; **Pursat** [1883–1885, *P.Couderc s.n.* (**P** P00093240); Phnom Kravanh, mont de Cardamomes, 29 November 1999, *S.Hul* et al. *797* (**P**-2 sheets); 18 December 2002, *S.Hul* et al. *1130* (**P**); Anlong Krauch, 100 m alt., 24 December 1968, *M.A.Martin 1330* (**P**-2 sheets)]; **Ratanak Kiri** [Sre Pok, 13°39′1″ N 106°56′34″ E, 104 m alt., 27 November 2007, *B.David* et al. *CL807* (**K**, **P**); Ban Tuh village, 250 m alt., 12 January 1969, *M.A.Martin 1475* (**P**-2 sheets)]; **Siem Reap** [Pres Chiane Dongrek, entre Samrong et Anlong Veng, 30 October 1927, *Poilane 13859* (**P**); Pres Chiane Dongrek, entre Comune Plouk, 4 November 1927, *Poilane 13941* (**AAU**, **BKF**, **P**)]. LAOS: **Luang Prabang** [Plantes du Louang Prabang, 1899, *C.Dupuy 121* (**P**-2 sheets); 300 m alt., 2 February 1970, *Pottier 628* (**P**); Phoutane, *Spire 146* (**P**); Plantes du Laos, *Spire 810* (**P**); Phoutane, *Spire 1524* (**P**); Phou Say, 29 December 1948, *J.E.Vidal 730B* (**P**)]; **Xiengkhuang** [Tran Ninh, environs de Xieng Khouang, September–December 1917, *Mieville 37192* (**P**); Tran Ninh, Tam Pa, 24 October 1920, *Poilane 2163* (**AAU**, **BKF**, **P**-2 sheets); Tran Ninh, Phak Lon, 5 September 1929, *Poilane 16876* (**P**); Plantes du Laos, *Spire 388* (**P**)]; **Vientiane** [Nong Thevada, 16 October 1955, *P.Tixier s.n.* (**P** P00093265); Environs Tha Ngon, 14 October 1965, *J.E.Vidal 4014* (**P**); Phou Khao Khouay, 800 m alt., 28 October 1971, *J.E.Vidal 5515* (**P**)]; **Khammouane** [Houay Wang Jang, 17°58′20″ N 105°24′17″ E, 596 m alt., 24 October 2005, *M.F.Newman* et al. *LAO 462* (**BKF**-2 sheets, **E**, **FOF**, **P**-2 sheets)]; **Savannakhet** [Entre Lao Bao et Muong Non, 18 April 1927, *Poilane 13290* (**P**); 13 October 1938, *Poilane 27028* (**AAU**, **BKF**, **P**-2 sheets); Entre Savannakhet et Pakse, 5 November 1938, *Poilane 28304* (**P**-2 sheets)]; **Champasack** [Bassac, expedition du Me Kong, 1866–1868, *Thorel s.n.* (**P** P00093264)]; **Attapeu** [Samakhizxai district, Sox village, 15°03′23.30” N, 106°59′08.10” E, 300 m alt., 19 December 2019, *P.Souladeth* et al. *L3488* (**FU**)]; **no province specified** [Laos, *M.Counillon s.n.* (**P** P00093248); Plantes du Laos, *M.Massie s.n.* (**P** P00093251)]. VIETNAM: **Tuyen Quan** [July 1885, *Brousmiche 336* (**P**)]; **Vinh Phuc** [Tam Dao, Huong Kahn, February 1909, *Ch. d’Alleizette 12* (**P**); Houng Kahn, October 1908, *Ch. d’Alleizette 126* (**P**); Nong Truong Tam Dao, 25 October 1976, *D.M.Thai 193D* (**HNU**); 25 October 1976, *D.M.Thai 0206D* (**HNU**); ibid., 25 October 1976, *D.M.Thai 0207D* (**HNU**)]; **Lang Son** [Lam Quan, 10 December 1902, *D.Bois 61* (**P**); Environs de Lang Son, 10 October 1902, *D.Bois 117* (**P**); Than Moi, *Eberhardt 3302* (**P**); Huu Lung, September 1962, *N.D.Khoi 092* (**HN**); ibid., September 1962, *N.D.Khoi 186* (**HN**-2 sheets); ibid., September 1962, *N.D.Khoi 187* (**HN**); ibid., 13 August 1962, *N.D.Khoi 1029* (**HNU**); ibid., August 1962, *N.D.Khoi 1029* (**HNU**); ibid., September 1962, *N.D.Khoi 1029* (**HNU**); Quan Ho, 20 October 1911, *H.Lecomte & A.Finet 2* (**P**); Cao Lang, Huu Lung, Song Hoa, 19 October 1974, *P.K.Loc P-1824* (**HNU**-2 sheets); Huu Lung, Hoa Thauy, 17 September 1963, *T.Vg s.n.* (**HNU**)]; **Quang Ninh** [Ouonbi, 12 September 1885, *B.Balansa 1253* (**P**); ibid., 12 November 1885, *B.Balansa 1254* (**P**); Quang Yen, Tankein, August 1885, *B.Balansa 1255* (**P**); Ouonbi, November 1885, *B.Balansa 1256* (**P**); Moncay, Tien Yen, Pointe Pagoda, 27 August 1932, *Rothe 47* (**P**)]; **Hai Duong** [Sept Pagodes, August 1908, *Mouret 39* (**P**)]; **Ha Nam** [Lat Son, 17 December 1885, *H.Bon 3097* (**P**-2 sheets)]; **Ninh Binh** [Dong-Giao, Cho Ganh, 8 October 1962, *Q.Hao 3161* (**HNU**-2 sheets); Cuc Phuong, 17 October 1963, *N.D.Khoi 306* (**HN**); Ngo Quan, Cuc Phuong, 17 October 1963, *N.D.Khoi s.n.* (**HN**); Dac Son, Bong Cuc Phuong, 27 December 1967, *Quy s.n.* (**HN**); Nga, Cuc Phuong, 4 September 1970, *Quy s.n.* (**HN**); Cho Ganh, September 1923, *Petelot 1032* (**P**); ibid., September 1923, *Petelot 1132* (**P**-2 sheets); ibid., October 1923, *Petelot 1142* (**HNU**, **P**-2 sheets); Dong-Giao, Cho Ganh, 8 September 1962 *S.N. 3161* (**HNU**)]; **Thanh Hoa** [Minh Son, Lang Din, 30 September 1962, *H.N.Dao 1029* (**HNU**)]; **Nghe An** [Quynchau, Carretera 1-Quynchau, 19°14′ N 105°36′ E, 100 m alt., 31 May 1993, *R.Schultze-Kraft* et al. *059/012-01* (**K**)]; **Quang Ngai** [Tam Quan, Quang Ngai-Tam Quan, 14°48′ N 108°58′ E, 80 m alt., 30 September 1993, *R.Schultze-Kraft* et al. *059/027-08* (**K**)]; **Kon Tum** [Dak Gley, ca. 7 km to south of Dak Gley town, near Dak Pet village, at 600–650 m alt. 12 November 1995, *L.Averyanov* et al. *VH 1517* (**AAU**, **HN**); Kon Plong, Tan Lap, 22 November 1978, *T.N.Ninh 130* (**HN**-3 sheets); 15 November 1978, *Phuong 642* (**HN**); Dak To, 1000 m alt., 23 September 1930, *Poilane 18367* (**P**); ibid., 600 m alt., 18 November 1947, *Poilane 35510* (**P**)]; **Binh Dinh** [Qui Nhon, Qui Nhon-Pleiku, 13°52′ N 108°58′ E, 150 m alt., 10 November 1993, *R.Schultze-Kraft* et al. *059/029-03* (**K**)]; **Gia Lai** [Chu Prong, 23 October 1978, *P.K.Loc & N.H.Ha P-2352* (**HNU**); Pleiku, Qui Nhon-Pleiku, 14°00′ N 108°13′ E, 700 m alt., 31 May 1993, *R.Schultze-Kraft* et al. *059/033-01* (**K**); An Khe, Gia Ma, 27 December 1977, *D.M.Thai 51* (**HNU**)]; **Dak Lak** [Cheo Reo, September, *J.Dournes s.n.* (**P** P00093287); Region de Ban Me Thuot, 400 m alt., 16 December 1948, *Schmid 753* (**P**); Ban Me Thuot, Ban Me Thuot-Ho Chi Minh, 12°33′ N 107°51′ E, 400 m alt., 22 July 1993, *R.Schultze-Kraft* et al. *059/038-03* (**K**)]; **Khanh Hoa** [Nha Trang et environs, 4–5 February 1914, *Chevalier 30445* (**P**-2 sheets); Nha Trang, region de Giang Che, 3 February 1922, *Poilane 2581* (**P**)]; **Dak Nong** [Duc Minh, 20 October 1980, *N.D.Khoi 640* (**HN**-2 sheets); Dak Mil-Duc Minh, 11 December 1979, *H.Tue 339* (**HN**-2 sheets)]; **Lam Dong** [Prenh pres Da Lat, 17 October 1924, *F.Evrard 1484* (**P**); Annam, Da Lat, Fimnon, 1000 m alt., October–November 1929, *R.Lichy 46* (**P**-2 sheets); Haut Dong Nai, Langanh, 1000–1200 m alt., 4 October 1932, *Poilane 21041* (**P**); Lang Hanh, 31 August 1967, *V. van Cuong 551* (**P**); Da Lat, Prenn, March 1967, *V. van Cuong 1103* (**P**)]; **Ninh Thuan** [Phan Rang, Ca Na, ca. 500 m alt., 23 November 1923, *Poilane 8685* (**AAU**, **P**); ibid., 800 m alt., 17 October 1925, *Poilane 12318* (**P**-2 sheets); Nui Chua, Nui Chua National Park, 546 m alt., 11.7066° N, 109.1380° E, 23 December 2023, *S.Tagane* et al. *N444* (**KAG**)]; **Binh Thuan** [Bac Binh, Phan Son Commune, 11°29′54″ N 108°20′39″ E, 863 m alt., 6 November 2013, *J.Leong-Skornickova* et al. *JLS 2667* (**P**)]; **Dong Nai** [Bien Hoa, Thu Duc, September 1865, *L.Pierre s.n.* (**E**, **P**-3 sheets P00093303, P00093304, and P03031073); Bien Hoa, 28 November 1928, *P. van Dieu 230* (**AAU**, **BKF**, **P**-2 sheets)]; **Tay Ninh** [Kedal, 7 January 1939, *Müller 741* (**P**)]; **Ba Ria-Vung Tau** [Cap. St. Jacques, 16 October 1919, *Poilane 551* (**P**-3 sheets); ibid., 18 October 1919, *Poilane 587* (**P**-3 sheets); Xuyen Moc, Binh Chau-Phuoc Buu reserved area, 10°32′49.7″ N 107°29′3.4″ E, 127 m alt., 3 October 2005, *N. van Du* et al. *HNK 1012* (**K**, **KEP**)]; **Ha Noi** [Dung Tien, 28 August 1963, *C.Dai s.n.* (**HNU**); Son Tay, Bavi, 570 m alt., 4 January 1965, *D. KS Viet-Trung s.n.* (**HN**-2 sheets)]; **Hai Phong** [Dao Cat Ba, 20 October 1975, *H.K.Nhue s.n.* (**HNU**); ibid., 23 October 1975, *H.K.Nhue s.n.* (**HNU**-3 sheets)]; **Ho Chi Minh** [Environs de Saigon, 30 October 1864, *E.Lefevre 267* (**P**)]; **Tonkin** [Tu Phap, 6 September 1886, *B.Balansa 2190* (**K**, **P**); Bach Bak, 14 September 1881, *H.Bon 766* (**P**-2 sheets)]; **Annam** [Yabak, 800–900 m alt., 2 October 1917, *Chevalier 35446* (**P**); 13 August 1939, *Müller 2047* (**P**)]; **Cochinchine** [1868, *Talmy s.n.* (**P** P00093320); 1862–1866, *Thorel 720* (**P**-2 sheets); 1862–1866, *Thorel s.n.* (**E**)]; **no province specified** [Trong Lou, 5 November 1865, *Brousmiche 315* (**P**); Marais de Gougah, December 1959, *P.Tixier s.n.* (**P** P00093323)].

**Conservation status**—CAMBODIA: This species is widespread in the country, with an EOO of 75,414.92 km^2^ and an AOO of 40 km^2^. It is assessed as LC. LAOS: This species is also common in Laos, with an EOO of 94,343.74 km^2^ and an AOO of 44 km^2^. Therefore, it is assessed as LC. VIETNAM: Multiple specimens were collected from various regions of Vietnam. The species has an EOO of 358,660.14 km^2^ and an AOO of 164 km^2^ and is assessed as LC. INDOCHINA: *P. pulchellum* is a widespread species in the region, and its conservation status must be classified as LC.

**5. *Phyllodium vestitum*** Benth. (1853: 217). Figure 1K,L and Figure 2B.

*Dicerma vestitum* Wall. (1831–1832: no. 5739). Type: Myanmar, *Wallich 5739* (holotype **K!**, isotypes **G**, n.v.-2 sheets).

*Desmodium vestitum* (Benth.) Benth. ex Baker (1879: 162).

Shrubs, 0.5–4 m tall with puberulous to pubescent hairy; stipules up to 5 mm long; petioles 0.5–2 mm long; rachis up to 3 cm long. *Leaflets* ovate to elliptic, 5–15 cm long, 2.5–8 cm wide. *Inflorescences* 0.2–1 m long, multi-branched; foliaceous primary bracts present; secondary bracts ovate, up to 1.5 mm long. *Flowers* 1.2–1.5 cm long, 5–8 per fascicle; bracteoles ovate, up to 1.2 mm long; pedicels 4–7 mm long; calyx tube 2–3 mm long; petals creamy white to green, 11–12 mm long; stamens up to 12 mm long. *Pods* 2–3 articulate, 11–15 mm long, outer surface reticulate, glabrescent to puberulous; articles suborbicular, ca. 5 mm long; seeds ca. 2 mm long, ca. 3 mm wide.

**Distribution**—Myanmar, Thailand, Cambodia, Laos, Vietnam, and Peninsula Malaysia.

**Ecology**—Mixed deciduous forests growing on sandy loam soil and bedrock area, 32–650 m alt.

**Phenology**—Flowering: October–April. Fruiting: August–February.

**Vernacular**—CAMBODIA: amprom pre kroi (Khmer-Kampong Chhnang, Kampong Thom); bay son lok (Khmer-Stung Treng); kantuy tra kuot (Khmer-Stung Treng); tronum bang kuoy (Khmer-Kampong Thom).

**Specimens examined**—CAMBODIA: **Kampong Chhnang** [Prey Chang Ka Tamau, 22 January 1918, *Chevalier 36950* (**P**)]; **Kampong Thom** [Bampe, Kampong Svai, 5 January 1920, *Bejaud 13* (**P**-2 sheets); 36 m alt., 24 November 2010, 12.3802° N 105.1629° E, *H.Toyama* et al. *391* (**FU**); 32 m alt., 24 November 2010, 12.3800° N 105.1618° E, *H.Toyama* et al. *396* (**FU**)]; **Stung Treng** [Siem Pang, Prek Meas, 7 February 2019, *S.Hul* et al. *7044* (**P**); Entre Anlong Veng et Slek Krei, 18 November 1927, *Poilane 14088* (**AAU**, **BKF**, **P**-2 sheets)]; **no province specified** [Cambodge, *Aubreville 25* (**P**)]. LAOS: **Khammouane** [Entre la Se Ban Fai et Takhet, 19 October 1938, *Poilane 28160* (**AAU**-2 sheets, **P**-2 sheets)]; **Savannakhet** [Xonbouly, Nonh Khu Nuea village, 16°23′48″ N 105°18.94′ 00″ E, 5 February 2000, *K.Sydara LAOS_098* (**P**)]; **Champasack** [Khong, Khinak village, 75 m alt., 2 February 1998, *J.F.Maxwell 98-108* (**CMUB**, **L**-2 sheets, **NUOL**); Bassac, expedition du Me-Kong, 9 August 1866–1868, *Thorel 2727* (**P**-3 sheets)]; **no province specified** [Indochina, southern Laos, Bassin du Se Lamphau, January 1876, *Harmand 205* (**P**-4 sheets)]. VIETNAM: **Dak Lak** [Annam, Ban Me Thout, *Schmid 4* (**P**); Sommet du Chu Keh, 650 m alt., December 1949, *M.Schmid 762* (**P**)]; **Ba Ria-Vung Tau** [Cape St. Jacques, *P.H.Ho 5110* (**P**); Ba Ria, April 1866, *L.Pierre s.n.* (**P** P00093350)]; **Ho Chi Minh** [Saigon, December 1865, *L.Pierre s.n.* (**P**-6 sheets, P00093344, P00093345, P00093346, P00093347, P00093348, and P00093349)].

**Conservation status**—CAMBODIA: Six specimens, representing five localities, were collected from the central and northern regions of Cambodia. The species has an EOO of 26,380.73 km^2^ and an AOO of 16 km^2^ and is listed as Near Threatened (NT) in the country. LAOS: Four specimens were collected from the central and southern regions of Laos. The species has a small range, with an EOO of 5825.82 km^2^, an AOO of 16 km^2^, and four collections; it is rated as VU: B2ab(iv) in the country. VIETNAM: This species is confined to southern Vietnam, with five collections. It has an EOO of 11,390.95 km^2^ and an AOO of 20 km^2^ and is classified as VU: B2ab(iv). INDOCHINA: This species is found from the central to southern parts of the region and is listed as LC.

## 3. Discussion

### 3.1. Classification and Characteristics

The genus *Phyllodium* is classified under the tribe Desmodieae based on the presence of an articulate pod, as noted by Hutchinson [5]. The present study confirms this classification, as all species of *Phyllodium* in the study area have articulate pods. This characteristic was also reported in all genera within the tribe, e.g., *Campylotropis* [26], *Codariocalyx* [27], *Dendrolobium* [28], *Pleurolobus*, and *Sohmaea* [29]. This suggests that the articulate pod may be a common feature of the tribe. This characteristic can support the phylogenetic analysis of Jabbour et al. [7]. At the genus level, the foliaceous primary bracts may serve as a key diagnostic feature of the genus, distinguishing it from other genera within the tribe. This characteristic has been previously mentioned in several studies, e.g., [8,9,10,21]. The genus was subsequently divided into two subgenera, *Phyllodium* and *Prainia*. Species classified under the subgenus *Phyllodium* possess a uniform primary bract, whereas species belonging to the subgenus *Prainia* exhibit a dimorphic primary bract [8]. Five species found in Cambodia, Laos, and Vietnam are members of the subgenus *Phyllodium*, as they have a uniform primary bract.

### 3.2. Diversity and Distribution in Cambodia, Laos, and Vietnam

The genus *Phyllodium* consists of seven species worldwide, including *P. elegans*, *P. hackeri*, *P. insigne*, *P. kurzianum*, *P. longpies*, *P. pulchellum*, and *P. vestitum* [8,9]. Thailand exhibits the highest species diversity, with six species [10]. Meanwhile Laos and Vietnam have five species each and Cambodia is home to four species. *Phyllodium pulchellum* is the most common species in these three countries. It has a widespread distribution across the Asian and Australian continents [8,11]. *Phyllodium kurzianum*, currently distributed in Laos and Vietnam, is likely to be found in Cambodia as well. This species has been recorded in the eastern part of Thailand, near northern Cambodia [10], and a few samples have been collected from southern Vietnam, which is also adjacent to eastern Cambodia. Further fieldwork and specimen collection in Cambodia may confirm the presence of this species. The present study suggests that *P. kurzianum* specimens from Laos should be added to the POWO [12] database to improve the accuracy of the species distribution data. The species distribution in this study is consistent with Dy Phon et al. [20], Newman et al. [17], and Jin et al. [18]. However, it differs slightly from the Indochina legume checklist [30], as *P. kurzianum* and *P. vestitum* were not previously recorded in Laos, and *P. pulchellum* was not reported in Cambodia.

### 3.3. Nomenclature and Typification

Two names, *Desmodium longipes* Craib and *D. tonkinense* Schindl., are lectotypified. The former name was published by Craib [31] based on three collections, *Godefroy* s.n., *Balansa* 1252, and *Kerr* 715. Dy Phon et al. [20] then chose the lectotype for *D. longipes* in K (*A.F.G. Kerr* 715). However, two duplicates of this gathering have been revealed at K. In accordance with the ICN rules and recommendations (Article 9.17), a second-step lectotype for this name must be designated [24]. Consequently, the duplicate with better preservation (K000587485) is herein selected as the second-step lectotype. *D. tonkinense* Schindl. was originally described by Schindler [32] based on nine specimen collections, *Balansa* 1252, *D’Alleizette* 14, *Godefroy* s.n., *Godefroy* s.n., *Harmand* s.n., *Lecomte & Finet* 336, *Pierre* s.n., *Simond* s.n., and *Spire* 407. Among these, a duplicate of *Balansa* 1252 (P00093141) has been designated as the lectotype, as it represents the most complete specimen, containing leaves, primary bracts, flowers, fruits, and a well-written label.

### 3.4. Ecology and Phenology

*Phyllodium elegans* is distinguished by its capacity to thrive in degraded habitats, the widest altitudinal range (30–2170 m), and a prolonged flowering and fruiting period of up to 10 months. Similarly, *P. longipes* and *P. pulchellum* are commonly found in open areas of deciduous to mixed deciduous forests at altitudes ranging from 15 to 1200 m, with extended flowering periods lasting 9 to 11 months. These characteristics may indicate a high level of resilience to environmental disturbances, broad ecological adaptability, and sustained reproductive success [33,34]. *P. kurzianum* has a broad altitude range (150–1010), which could facilitate its adaptability. However, its shorter flowering period suggests that reproductive success may rely on other factors, e.g., pollination efficiency or breeding system [35]. *P. vestitum*, with a more limited altitude range (32–650 m), may have a narrower ecological niche. This could make it more sensitive to environmental changes or disturbances compared to the more adaptable species [36].

### 3.5. Conservation Status

Among the five species, *P. elegans* and *P. pulchellum* consistently maintain a conservation status of LC across Cambodia, Laos, Vietnam, and the broader Indochina region. This classification is primarily attributed to their wide geographic distribution and relatively large EOO and AOO values, particularly in Vietnam and Indochina. In contrast, *P. kurzianum*, *P. longipes*, and *P. vestitum* demonstrate varying degrees of threat across their ranges. *P. kurzianum* is primarily classified as EN in Laos due to its extremely restricted distribution (EOO: 206.73 km^2^; AOO: 12 km^2^) and presence at a limited number of locations. From an ecological perspective, this species demonstrates adaptability across a broad altitudinal gradient (150−1010 m above sea level); the species is then assessed as DD instead, while it is assessed as NT in Vietnam. Similarly, *P. longipes* is listed as EN in Cambodia, reflecting its very small EOO (46.41 km^2^) and AOO (12 km^2^), although it is categorized as LC in both Laos and Vietnam. *P. vestitum* represents a species under consistent pressure across its range; it is designated as NT in Cambodia and VU in both Laos and Vietnam, due to its limited EOO and AOO values and its occurrence in fewer than ten locations. The conservation status of the *Phyllodium* species remains largely undocumented at national, regional, and global scales. However, two species, *P. elegans* and *P. pulchellum*, have been evaluated at the global scale and classified as LC due to their broad distribution and abundance within their natural habitats [37,38]. Additionally, *P. pulchellum* has been assessed as NT for Sri Lanka [39]. Since the time of the early collections (19th to mid-20th century), the Indochina region has experienced major socio-political and environmental changes, including armed conflicts, increased human activity, and significant land use changes. These disturbances have likely affected local ecosystems, influencing habitat structure, species composition, and ecological functions. Although detailed information is lacking, it is likely that the conservation status of the *Phyllodium* species has been impacted. Therefore, it is important to investigate how these long-term pressures have influenced population survival, habitat availability, and ecological relationships.

## 4. Materials and Methods

The study of the genus *Phyllodium* in Cambodia, Laos, and Vietnam is based on field collections and herbarium specimens from AAU, ABD, BKF, BM, C, CMUB, E, FOF, FU, G, G-DC, HN, HNU, K, KAG, KEP, L, NUOL, P, QBG, and SING. In addition, online photographs of herbarium specimens from GH and BR are also studied. Herbarium acronyms are based on Thiers [40], and the herbarium of the Department of Biology, Faculty of Natural Science, National University of Laos, is consistently referred to as NUOL throughout this paper. This study provides complete taxonomic information for the genus and species of Cambodia, Laos, and Vietnam. The information on the original publication of each name can be found in a taxonomic treatment of Saisorn and Chantaranothai [10]. It also covers the distribution, ecology, phenology, vernacular names, specimens examined, and photographs. The distribution map of each species is drawn using QGIS software version 3.34.14 [41]. The conservation status assessment at regional and national levels is based on the IUCN [42] and the IUCN Standards and Petitions Committee [43]. An Extend of Occurrence (EOO) and an Area of Occupancy (AOO) are calculated by a Geospatial Conservation Assessment Tool (GeoCAT) version BETA with a default cell width (2 km) [44]. The lectotypification has been made following the ICN [24].

## 5. Conclusions

This study provides a comprehensive taxonomic study of the genus *Phyllodium* in Cambodia, Laos, and Vietnam. Five species of *Phyllodium* are recognized from this area. Of these, five species from Laos and Vietnam and four species from Cambodia are recorded. Second-step lectotypification of *D. longipes* Craib and lectotypification of *D. tonkinense* Schindl. are made, and the conservation status assessment is discussed. Our findings serve as the updated knowledge of the genus *Phyllodium* for the Flora of Cambodia, Laos, and Vietnam.

## Data Availability

The data are contained within the article.

## References

[1] Desvaux N.A. (1813). Précis des caractéres de plusieurs genres de la famille des Légumineuses. J. Bot. Agric..

[2] De Candolle A.P. (1825). Sive ordines, genera et species plantarum secundum methodi naturalis normas digestarum et descriptarum. Regni Veg. Syst. Nat..

[3] Bentham G., Hook F. (1865). Sistens dicotyledonum polypetalarum ordines XI: Leguminosas-Myrtaceas. Gen. Plant..

[4] Baker J.G., Hooker J.D. (1879). Leguminosae. The Flora of British India.

[5] Hutchinson J. (1964). The Genera of Flowering Plants (Angiospermae): Dicotyledones.

[6] Ohashi H., Lewis G., Schrire B., Mackinder B., Lock M. (2005). Tribe Desmodieae. Legumes of the World.

[7] Jabbour F., Gaudeul M., Lambourdière J., Ramstein G., Hassanin A., Labat J.-N., Sarthou C. (2018). Phylogeny, biogeography and character evolution in the tribe Desmodieae (Fabaceae: Papilionoideae), with special emphasis on the New Caledonian endemic genera. Mol. Phyl. Evol..

[8] Ohashi H. (1973). The Asiatic species of *Desmodium* and its allied genera (Leguminosae). Ginkgoana.

[9] Pedley L. (1999). *Desmodium* Desv. (Fabaceae) and related genera in Australia: A taxonomic revision. Austrobaileya.

[10] Saisorn W., Chantaranothai P. (2015). Taxonomic studies on the genus *Phyllodium* Desv. (Leguminosae) in Thailand. Trop. Nat. Hist..

[11] Ohashi H. (2004). Taxonomy and distribution of *Desmodium* and related genera (Leguminosae) in Malesia (II). J. Jpn. Bot..

[12] Plants of the World Online (POWO). https://powo.science.kew.org/.

[13] Sanjappa M. (1992). Legumes of India.

[14] Pedley L., Dassanayake M.D., Clayton W.D. (1996). Tribe Desmodieae. A Revised Handbook to the Flora of Ceylon.

[15] Grierson A.J.C., Long D.G. (1987). Family 76. Leguminosae. Flora of Bhutan.

[16] Huang P.H., Ohashi H., Wu Z.Y., Raven P.H., Hong D.Y. (2010). Phyllodium Desvaux. Flora of China.

[17] Newman M., Ketphanh S., Svengsuksa B., Thomas P., Sengdala K., Lamxay V., Armstrong K. (2007). A Checklist of the Vascular Plants in Lao PDR.

[18] Jin H.-Y., Ahn T.-H., Lee H.J., Song J.H., Lee C.H., Kim Y.J., Yoon J.W., Chamg K.S., Kang H.S., Cheng H.C. (2016). A Checklist of Plants in Laos PDR.

[19] Merrill E.D. (1923). An Enumeration of Philippine Flowering Plants.

[20] Dy Phon P., Ohashi H., Vidal J.E., Morat P. (1994). Leguminosae (Fabaceae) Papilionoideae Desmodieae. Flore du Cambodge du Laos et du Viêtnam.

[21] Ridley H.N. (1922). Flora of the Malay Peninsula vol. 1–Polypetalae.

[22] Backer C.A., van den Brink R.C.B. (1963). Flora of Java (Spermatophytes Only).

[23] Verdcourt B. (1979). A Manual of New Guinea Legumes.

[24] Turland N.J., Wiersema J.H., Barrie F.R., Greuter W., Hawksworth D.L., Herendeen P.S., Knapp S., Kusber W.-H., Li D.-Z., Marhold K. (2018). International Code of Nomenclature for Algae, Fungi, and Plants (Shenzhen Code) Adopted by the Nineteenth International Botanical Congress Shenzhen, China, July 2017.

[25] Ohashi H., Ohashi K., Adema A.C.B. (2017). Lectotype of *Phyllodium elegans* var. *javanicum* (Leguminosae/Fabaceae tribe Desmodieae). J. Jpn. Bot..

[26] Satthaphorn J., Roongsattham P., Chantaranothai P., Leeratiwong C. (2018). The genus *Campylotropis* (Leguminosae) in Thailand. Thai For. Bull..

[27] Saisorn W., Chantaranothai P. (2019). The genus *Codariocalyx* Hassk. (Leguminosae-Papilionoideae) in Thailand: Taxonomy and anatomy of leaf and stem. Songklanakarin J. Sci. Technol..

[28] Saisorn W., Chantaranothai P. (2021). Taxonomy of *Dendrolobium* (Leguminosae) in Thailand. Trop. Nat. Hist..

[29] Saisorn W., Chantaranothai P. (2022). A taxonomic revision of two genera, *Pleurolobus* and *Sohmaea* (Leguminosae) in Thailand and Indo-China. Phytotaxa.

[30] Lock J.M., Heald J. (1994). Legumes of Indo-China: A Check-List.

[31] Craib W.G. (1910). IV.−Decades Kewenses: Plantarum Novarum in herbario horti regii conservation. Bull. Misc. Inform. Kew.

[32] Schindler A.K. (1916). Desmodiinae novae. Bot. Jahrb. Syst..

[33] Rodríguez-Pérez J., Traveset A. (2016). Effect of flowering phenology and synchrony on the reproductive success of a long-flowering shrub. AoB Plants.

[34] Yaqoob U., Nawchoo I.A. (2017). Impact of habitat variability and altitude on growth dynamics and reproductive allocation in *Ferula jaeschkeana* Vatke. J. King Saud. Univ.-Sci..

[35] Ma Y., Yin G., Gao J., Luo Y.-B., Bai W.-N. (2019). Effects of distinct pollinators on the mating system and reproductive success in *Incarvillea sinensis*, an annual with large floral displays. J. Plant. Ecol..

[36] Schweiger O., Heikkinen R.K., Harpke A., Hickler T., Klotz S., Kudrna O., Kuhn I., Poyry J., Settele J. (2012). Increasing range mismatching of interacting species under global change is related to their ecological characteristics. Glob. Ecol. Biogeogr..

[37] Contu S. (2012). *Phyllodium elegans*. The IUCN Red List of Threatened Species 2012: E.T19891571A20127741. https://www.iucnredlist.org/species/19891571/20127741.

[38] Poveda L.L. (2012). *Phyllodium pulchellum*. The IUCN Red List of Threatened Species 2012: E.T19891445A20128016. https://www.iucnredlist.org/species/19891445/20128016.

[39] Wijesundara S., Ranasinghe S., Jayasinghe H., Gunawardena N., Fonseka G., Wijesooriya S. (2020). Angiosperms in Sri Lanka: Present status of Angiosperms in Sri Lanka. The National Red List 2020—Conservation Status of the Flora of Sri Lanka.

[40] Thiers B.M. (Updated Continuously). Index Herbariorum. https://sweetgum.nybg.org/science/ih/.

[41] QGIS Development Team (2023). QGIS Geographic Information System (QGIS).

[42] IUCN (2012). Guidelines for Application of IUCN Red List Criteria at Regional and National Levels.

[43] IUCN Standards and Petitions Committee (2024). Guidelines for Using the IUCN Red List Categories and Criteria.

[44] Bachman S., Moat J., Hill A.W., de la Torre J., Scott B. (2011). Supporting Red List Threat Assessments with GeoCAT: Geospatial Conservation Assessment Tool. ZooKeys.

